# From silos to synergy: trainee responses to multidisciplinary training in child and adolescent psychiatry

**DOI:** 10.3389/fpsyt.2025.1679862

**Published:** 2025-11-07

**Authors:** Masahide Usami, Yuki Mizumoto, Kotoe Itagaki, Keita Yamamoto, Kozo Ohcho, Masahiro Fukuda, Ayaka Taniguchi, Masakage Okuno, Noa Tsujii, Yuzuru Harada, Takashi Nagasawa, Kazuhiko Saito

**Affiliations:** 1 Department of Child and Adolescent Psychiatry, National Kohnodai Medical Center, Japan Institute for Health Security, Ichikawa, Japan; 2 Department of Clinical Psychology, National Kohnodai Medical Center, Japan Institute for Health Security, Ichikawa, Japan; 3 Department of Social Work, National Kohnodai Medical Center, Japan Institute for Health Security, Ichikawa, Japan; 4 Department of Psychiatry, Okayama Psychiatric Medical Center, Okayama, Japan; 5 Department of Clinical Psychology, Okayama Psychiatric Medical Center, Okayama, Japan; 6 Department of Mental Health Social Work, Center for Psychosocial Independence, Okayama Psychiatric Medical Center, Okayama, Japan; 7 Mikuni-hill Mental Clinic, Mikuni-hill Hospital, Osaka, Japan; 8 Department of Child Mental Health and Development, Toyama University Hospital, Toyama, Japan; 9 Department of Child and Adolescent Psychiatry, Nagano Prefectural Mental Wellness Center, Komagane, Nagano, Japan; 10 Department of Child and Adolescent Psychiatry, Tokyo Metropolitan Children’s Medical Center, Tokyo, Japan; 11 Aiiku Research Institute, Imperial Gift Foundation Boshi-Aiiku-Kai, Tokyo, Japan

**Keywords:** child and adolescent psychiatry, multidisciplinary training, interprofessional education, net promoter score, educational program evaluation, factor analysis, Japan

## Abstract

**Introduction:**

Japan is experiencing a growing demand for child and adolescent mental health services owing to the increasing rates of school refusal, youth suicide, and neurodevelopmental disorders. However, there is a critical shortage of trained professionals as well as limited national efforts to provide multidisciplinary training.

**Methods:**

We evaluated a government-funded training program aimed at enhancing collaboration between clinical, educational, and psychosocial professionals in child psychiatry. A total of 426 participants completed post-training surveys assessing the perceived usefulness of 22 lecture modules (rated 0–10), overall satisfaction (rated 0–10), and open-ended feedback on the curriculum design. An exploratory factor analysis was conducted to identify the latent domains among the modules. Net promoter scores (NPSs) were calculated for each module to gauge the perceived value. Ordinary least-squares regression was used to identify the predictors of overall satisfaction.

**Results:**

Four content domains were identified: foundational knowledge, therapeutic skills, problem behavior management, and multidisciplinary collaboration. The mean satisfaction score was 4.6/5. The modules with the highest NPS were team-based care and practical case-based learning. Participants with educational and psychosocial roles reported significantly higher satisfaction than clinical staff. The regression analysis revealed that therapeutic skills and collaboration were significant predictors of overall satisfaction.

**Conclusion:**

Multidisciplinary training programs are feasible and well-received in Japan. The participants prioritized interactive, role-based learning and emphasized the need for content tailored to real-world teamwork. These findings could inform future curriculum development and workforce policies in child and adolescent psychiatry.

## 1 Introduction

Child and adolescent psychiatry addresses the complex interplay among developmental, psychological, familial, and educational factors that influence youth mental health. Effective care for this population requires multidisciplinary collaboration among child psychiatrists, clinical psychologists, nurses, social workers, and educators (1, 2). In Japan, recent policy reforms and epidemiological shifts, such as an increase in school refusal, neurodevelopmental disorders, and family dysfunction, have highlighted the urgent need for integrated support systems (3, 4).

In Japan, child and adolescent mental health is facing a mounting crisis characterized by multiple converging factors. In 2022, over 300, 000 children were reported as school refusers, an all-time high, while youth suicide remained the leading cause of death among those aged 10–19 years. These alarming trends, exacerbated by the coronavirus disease 2019 pandemic, academic pressure, family dysfunction, and social isolation, underscore the urgent need for comprehensive mental health support (5–8).

Similarly, neurodevelopmental disorders, especially autism spectrum disorder (ASD) and attention-deficit/hyperactivity disorder (ADHD), are increasingly being diagnosed (9–11). According to the Ministry of Health, Labor, and Welfare (MHLW), the number of children receiving treatment for developmental disorders has tripled over the past decade.

However, serious workforce shortages persist. Fewer than 500 board-certified child and adolescent psychiatrists cater to over 16 million youths, with services concentrated in urban areas (4, 12). This mismatch between the rising clinical demand and limited professional capacity highlights the urgent need to develop scalable multidisciplinary training programs nationwide.

International evidence has demonstrated that integrated team-based care enhances diagnostic accuracy, improves treatment adherence, and increases family satisfaction. However, in Japan, training remains largely siloed to professions, and interprofessional educational opportunities are rare, especially outside metropolitan areas (12).

In response to growing clinical demands and policy shifts, the MHLW initiated the “Development of a Manual to Promote Multidisciplinary Collaboration in Child and Adolescent Psychiatry” project in 2021.

From the fiscal year (FY)2017 to FY2021, a national training program delivered 16 foundational lectures in both basic and advanced formats. However, in FY2023, six new modules were added to the curriculum: cognitive behavioral therapy (CBT), play therapy, electroconvulsive therapy (ECT), and three inpatient-focused lectures covering the roles of psychiatric nurses, clinical psychologists, and social workers. These were introduced with reference to the established adult psychiatry training programs at the National Center of Neurology and Psychiatry (NCNP), where such modules are commonly included (13, 14).

Importantly, this revision was timed with the FY2024 national insurance policy update, which recognizes training for allied health professionals (including nurses, psychologists, and social workers) as reimbursable when providing team-based care. This alignment with insurance incentives highlights the growing emphasis on comprehensive multidisciplinary competencies in child psychiatry.

The revised 22-module structure was retrospectively validated through a nationwide needs assessment survey of 1, 240 past trainees, identifying essential modules from the perspective of diverse professionals, resulting in the 2024 pilot training comprising 53 new participants.

Until FY2021, Japan’s national training program for child and adolescent mental health consisted of two sequential courses: a basic and an advanced program. The basic program was initially conducted entirely in person; however, in 2021, it transitioned to a fully online format, combining live-streamed and on-demand lectures. The 2-day structure of the advanced program was maintained; however, in-person small-group case supervision sessions were introduced on day 2.

Both courses follow a shared curriculum comprising 16 core lectures that collectively provide a comprehensive foundation for child and adolescent psychiatry. These lectures addressed (1) national mental health policy (2); ADHD (3); ASD (4); schizophrenia (5); mood disorders (6); anxiety disorders (7); obsessive–compulsive disorder (8); internet addiction (9); self-harm and suicide prevention (10); eating disorders (11); somatic symptom disorder (12); child abuse and its care (13); psychopharmacology (14); inpatient treatment (15); family therapy; and (16) group therapy.

In the advanced program, participants were organized into small interdisciplinary teams, with each trainee presenting one of four de-identified clinical cases. Through guided group discussions and expert feedback, these sessions allowed participants to apply the lecture content to real-world situations and enhance their collaborative clinical reasoning skills.

Therefore, this study aimed to evaluate the content structure and perceived utility of a nationwide multidisciplinary training program in child and adolescent psychiatry in Japan. Using exploratory factor analysis, we sought to identify latent dimensions underlying participant evaluations of 22 standard training modules. We further examined the predictors of overall training satisfaction, focusing on content domains and professional background.

We hypothesized that specific training domains—particularly those related to therapeutic skills and multidisciplinary collaboration—would be more strongly associated with satisfaction than others. We also expected differences in perceived utility across professional subgroups. These findings are intended to inform the design of future interdisciplinary training initiatives and workforce development strategies in mental health care for youth.

## 2 Materials and methods

### 2.1 Study design and participants

The revised training program included 22 lectures, of which six were newly introduced in 2024 to address both clinical gaps and upcoming insurance criteria for allied health professionals.

Between October 2023 and March 2024, 1, 240 professionals who participated in MHLW-commissioned adolescent mental health Training between FY2017 and FY2023 were invited via email to complete an online survey. A total of 426 respondents (response rate of 34%) completed the questionnaire and provided informed consent.

### 2.2 Online survey

#### 2.2.1 Evaluation of training content

The items evaluated in the lectures were, in general, questions about child psychiatric examinations, systematic lectures on specific disorders, psychological testing and evaluation, general child welfare in the community, mental health and welfare laws, and team-based medical care in child psychiatry. The treatment section included questions on child psychotherapy, pharmacotherapy in child psychiatry, family therapy, CBT, group psychotherapy, sleep hygiene guidance, play therapy, ECT, and inpatient treatment in child psychiatry. The problematic behavior section included questions on truancy/withdrawal, self-harm/suicide, delinquency, domestic violence, and child abuse. In the occupational therapy category, questions were asked about nursing and full-time staff duties (psychologists, mental health welfare workers, and social workers) in child psychiatric inpatient treatment.

For each lecture, the respondents were asked about the training that people in the same occupation should receive when starting to practice in child psychiatry. However, the lectures were assumed to last 50 min, given by experts in each field, either online or in person. The terms “children” and “childhood and adolescence” were defined as those aged <18 years.

We obtained responses using a 10-point scale (0–10 points) based on the evaluation of each lecture conducted in the MHLW Mental Health Promotion Program for Children and Adolescents. A score of 10 was defined as “essential for our job type, “ and a score of 1 was defined as “unnecessary for our job type.” Furthermore, this score was used to calculate the net promoter score (NPS) using the following formula:

NPS = [(number of promoters, 9–10) – (number of detractors, 1–6)] / total respondents × 100.

This rating was intended to assess the perceived practical relevance of each module to the respondents’ professional duties, rather than to directly measure skill acquisition or educational effectiveness.

The results are presented by NPS. An NPS of ≥0 was considered good, ≥20 was desirable, > 50 was excellent, and >80 was a top-level recommendation (15–18).

#### 2.2.2 Additional training

In addition to the lecture curriculum of the MHLW Mental Health Promotion Program for Adolescents, which is currently being implemented, we also asked about the disease-specific lecture content that should be provided to new employees in the same occupations as the respondents.

#### 2.2.3 Child and adolescent psychiatric care setting

Regarding the approach to training in child and adolescent psychiatric care, it was specified that the training should be a 50-min lecture delivered by experts in each field via online or in-person sessions. Regarding the methods of training that individuals in the same profession as the respondents must undergo when practicing child and adolescent psychiatric care as newcomers, the respondents were asked to choose from the following options: acquiring basic knowledge through on-demand videos, acquiring basic knowledge through in-person lectures, receiving practical guidance through clinical training at hospitals, improving understanding through multidisciplinary case discussions, presenting at academic conferences, individual case supervision, and individual case supervision.

#### 2.2.4 Multidisciplinary collaboration

Respondents were asked about the frequency and participating professions of regular multidisciplinary meetings held within their facilities (such as multidisciplinary discharge support meetings, stakeholder meetings, and ward case conferences). The professionals included child psychiatrists, psychiatrists, pediatricians, resident physicians, physicians (other than the above), nurses/public health nurses, licensed psychologists/clinical psychologists, school counselors, mental health welfare workers, social welfare workers, and educators such as teachers. Additionally, respondents were asked to identify the challenges of interprofessional collaboration and select up to three professions that they found the most difficult, choosing from the following options: “I feel difficulties almost every day, I occasionally feel difficulties, “ “I rarely feel difficulties, “ and “I hardly ever feel difficulties.

#### 2.2.5 Need for follow-up programs

Respondents were asked about the timing and frequency of conducting similar training related to child psychiatry since the initial training. The questions were designed such that the respondents could choose one option from the following: once a year, once every 2 years, once every 3 years, once every 4 years, once every 5 years, or not necessary.

### 2.3 Professional subgroups

To examine whether training outcomes varied across professional backgrounds, the participants were grouped into three broad categories based on their primary work context and functional roles in child and adolescent mental healthcare:

- Clinical care (including psychiatrists, clinical psychologists, and nurses),
- Educational professionals (including school staff and child welfare practitioners)
- Psychosocial support (including social workers).

This three-group classification was based on prior research and global policy frameworks that recognize that multidisciplinary teams in child and adolescent mental health commonly operate within three core functional domains: clinical care, education, and psychosocial support (2, 19). This function-based grouping improved interpretability and reflected real-world collaboration patterns among professionals. Such domain-based categorization improves the interpretability of subgroup analyses, especially when sample sizes within each original profession are limited.

### 2.4 Six components of the online survey

The questionnaire comprised six components:

1. Demographics and professional background: profession, years of clinical experience, and sex.
2. Lecture-module ratings: the 22 modules were rated from 0 (not useful) to 10 (absolutely essential).
3. Overall satisfaction: single-item rating (0–10).
4. Collaboration practices: frequency and composition of multidisciplinary meetings; perceived difficulty and barriers.
5. Follow-up program: preferred interval for refresher training.
6. Open-ended feedback: suggestions for curriculum improvements.

### 2.5 Statistical analysis

Descriptive statistics were used to summarize respondent characteristics and module-level NPS. Exploratory factor analysis (EFA) was conducted to identify clusters of lecture modules sharing similar patterns of perceived relevance across respondents. We used principal axis factoring with varimax rotation. EFA using maximum likelihood estimation was performed on 22 lecture module ratings (N = 426) to identify latent content domains. The EFA grouped modules into four domains reflecting underlying dimensions of perceived training relevance, providing a conceptual structure for interpreting patterns of trainee evaluations.

Four factors were extracted: foundational knowledge (F1), therapeutic skills (F2), problem behavior management (F3), and multidisciplinary collaboration (F4), based on eigenvalues > 1.0 and scree plot inspection.

A one-way analysis of variance (ANOVA) with Bonferroni-corrected *post hoc* tests was conducted to compare factor scores across the three professional subgroups: clinical care, educational professionals, and psychosocial support.

Overall satisfaction (0–10) was modeled using ordinary least squares (OLS) regression in the R software for inference. The independent variables included four-factor scores (F1–F4) and years of clinical experience. Assumption checks for OLS were conducted as follows:

- Multicollinearity was evaluated using variance inflation factors (VIF), all < 2.0.
- Normality of residuals was tested using the Shapiro–Wilk test.
- Homoscedasticity was assessed using the Breusch–Pagan test.

All statistical analyses were performed using R (version 4) and Python (version 3).

### 2.6 Ethical considerations

This study did not involve interventions or interactions with individuals beyond the scope of routine professional activities and thus falls outside the definition of research requiring formal ethical approval under Japanese regulations. The surveys were conducted as part of regular quality improvement and training development activities in child and adolescent psychiatric services.

All data were collected in a non-identifiable format. Before data collection, the participants were informed that their responses would be anonymized and could be used for research dissemination and publication. Consent was obtained through voluntary participation.

## 3 Results

### 3.1 Demographics and professional background

Of the 1, 240 invited trainees, 426 completed the survey (response rate: 34%) with a mean clinical experience of 6.2 years (SD = 6.8; median = 3 years), 73.8% female (n = 315), 25.4% male (n = 108), and 1% others (n = 4). The respondents included 92 clinical psychologists (21.5%), 70 nurses (16.4%), 69 public health nurses (16.1%), 62 social workers (14.5%), 43 psychiatrists (10.1%), 36 pediatricians (8.4%), nine occupational therapists (2.1%), nine child psychiatrists (2.1%), and 15 others (3.5%).

The professionals that reported difficulties included child psychiatrists, psychiatrists, pediatricians, junior resident physicians, physicians (other than the above), nurses, public health nurses, licensed psychologists/clinical psychologists, school counselors, mental health welfare workers,/social welfare workers, teachers, and other education professionals, and child guidance center staff.

### 3.2 Lecture‐module ratings

Participants rated the 22 modules from 0 to 10, followed by conversion to NPS. An overview of module ratings by domain is provided in **Tables 1**–**3**.

**Table 1:** Foundational knowledge (F1).

Professional subgroup	N	Child psychiatric examination	Disorder-specific lectures	Psychological testing and assessment	Community child welfare	Mental health and welfare law
Clinical care	346	8.35 ± 2.07	9.14 ± 1.57	8.45 ± 2.03	8.47 ± 1.74	8.37 ± 1.98
Education	18	7.83 ± 2.63	8.56 ± 3.00	8.56 ± 2.48	8.44 ± 2.36	7.50 ± 2.43
Psychosocial Support	62	8.05 ± 2.41	8.63 ± 2.00	7.61 ± 2.36	8.90 ± 1.67	8.98 ± 2.04
Total	426	8.29 ± 2.15	9.04 ± 1.74	8.33 ± 2.12	8.53 ± 1.77	8.42 ± 2.03

Mean ratings (M ± SD) by professional subgroup (0–10 scale).

**Table 2:** Therapeutic skills (F2).

Professional subgroup	N	Child psychotherapy	Pharmacotherapy in child psychiatry	Family therapy	Cognitive and behavioral therapy	Group psychotherapy	Sleep hygiene education	Play therapy	Electroconvulsive therapy
Clinical care	346	8.63 ± 1.91	8.40 ± 1.88	8.67 ± 1.74	8.27 ± 1.88	7.95 ± 2.02	7.96 ± 1.92	7.69 ± 2.21	5.90 ± 2.65
Education	18	7.61 ± 2.41	7.50 ± 2.61	8.06 ± 2.41	7.67 ± 2.54	6.17 ± 2.71	7.06 ± 3.06	7.83 ± 2.57	5.00 ± 3.11
Psychosocial Support	62	8.31 ± 1.82	7.87 ± 1.99	8.61 ± 1.78	8.03 ± 1.91	8.13 ± 1.88	7.15 ± 2.31	6.84 ± 2.27	6.47 ± 2.23
Total	426	8.54 ± 1.93	8.29 ± 1.95	8.63 ± 1.78	8.21 ± 1.92	7.90 ± 2.07	7.80 ± 2.07	7.57 ± 2.26	5.94 ± 2.63

Mean ratings (M ± SD) by professional subgroup.

**Table 3:** Problem behavior management (F3).

Professional subgroup	N	School refusal	Self-harm and suicide	Delinquency and conduct disorders	Domestic violence	Child abuse
Clinical care	346	9.29 ± 1.30	9.38 ± 1.24	8.79 ± 1.69	8.92 ± 1.53	9.25 ± 1.38
Education	18	9.11 ± 1.88	8.94 ± 1.90	9.17 ± 1.92	8.83 ± 2.46	9.44 ± 2.29
Psychosocial Support	62	9.13 ± 1.52	8.97 ± 1.81	8.65 ± 1.70	8.85 ± 1.60	8.95 ± 1.95
Total	426	9.26 ± 1.37	9.30 ± 1.38	8.78 ± 1.70	8.91 ± 1.59	9.21 ± 1.53

Mean ratings (M ± SD) by professional subgroup.

- Foundational knowledge (six modules): All achieved NPSs ≥ 20. “Disorder‐specific lectures” scored the highest at NPS = 64, followed by “child welfare law” at 42 and “mental health welfare law” at 40. The lowest score in this domain, 36, was scored by “psychiatric examination.” Ratings for modules in the foundational domain are presented in **Table 1**.
- Therapeutic skills (nine modules): “Family therapy” (NPS = 49) and “pharmacotherapy” (36) were well-received; “CBT” (32) and “group psychotherapy” (20) fell into the “moderate” range. “sleep hygiene education” (15), “play therapy” (9), and notably “ECT” (–42) indicated significant dissatisfaction. Details for the therapeutic-skills domain are shown in **Table 2**.
- Problem‐behavior management (five modules): all exceeded NPS = 50. “Self‐harm and suicide prevention” led at 76, “school refusal and withdrawal” at 73, “child abuse” at 71, “domestic violence” at 58, and “delinquency and conduct disorders” led at 53. Evaluation results for the problem-behavior management domain are summarized in **Table 3**.
- Role‐specific topics (two modules): “Inpatient nursing” scored NPS = 6, and “dedicated psychology/welfare roles” scored 26, reflecting clear calls for enhanced collaborative practice training.

### 3.3 Overall satisfaction

Overall satisfaction was rated on a scale of 0 to 10 (M = 7.4, SD = 1.6). A total of 312 respondents (73.1%) rated their satisfaction ≥ 8, 85 (19.9%) rated between 5 and 7, and 30 (7.0%) rated ≤ 4, indicating broad approval of the training with room for improvement.

### 3.4 Collaboration practices

Among the 382 respondents who attended multidisciplinary meetings, 122 (31.9%) reported no regular meetings, 82 (21.5%) met once monthly, 31 (8.1%) met twice monthly, 11 (2.9%) met three times monthly, 99 (25.9%) met four or more times monthly, and 10 (2.6%) attended daily.

- Attendance: 159 nurses/public health nurses (37.2%) and 105 clinical psychologists (24.6%) “always” attend, whereas 117 child psychiatrists (27.4%) and 85 psychiatrists (19.9%) “never” attended multidisciplinary meetings.
- Perceived difficulty: 141 (33.0%) “sometimes” and 118 (27.6%) “occasionally” found collaboration challenging. The top barriers were a lack of information sharing (20; 4.7%), insufficient interagency cooperation (19; 4.4%), and resource constraints (13; 3.0%).

### 3.5 Follow-up programs

The preferred intervals for refresher training among the 426 respondents were as follows: 244 (57.1%) respondents selected annually, 62 (14.5%) every 2 years, 53 (12.4%) every 3 years, one (0.2%) every 4 years, 13 (3.0%) every 5 years, and five (1.2%) selected no further training was needed. Forty-nine patients (11.5%) did not respond.

### 3.6 Open-ended feedback

The analysis of 312 free‐text comments revealed five recurrent themes:

- Case-based learning (65%): Desire for regular, real-case multidisciplinary conferences.
- Simulation and role-play (58%): The need for hands-on workshops on practicing therapeutic techniques and team communication.
- Hybrid delivery (72%): Support for e-learning of theoretical aspects with in-person skill practice.
- Iterative feedback (47%): Emphasis on structured post-module evaluations and focus groups for curriculum refinement.
- Expanded topics (53%): Requests for modules on trauma-informed care (including trauma-focused CBT), school-based interventions, and family systems approaches.

These detailed quantitative and qualitative findings form a comprehensive evidence base guide for redesigning multidisciplinary training in child and adolescent psychiatry in Japan.

### 3.7 Predictors of overall satisfaction

To identify the training domains and participant characteristics that most strongly influenced overall satisfaction, we fitted an OLS regression model using the 0–10 satisfaction rating as the dependent variable. The independent variables were factor scores from the four identified content domains: foundational knowledge (F1), therapeutic skills (F2), problem behavior management (F3), and multidisciplinary collaboration (F4); and years of clinical experience. Analysis was conducted using a subset of 420 respondents with complete data. Estimated coefficients with 95% confidence intervals are reported in **Table 4**.

**Table 4:** Predictors of overall satisfaction (OLS regression).

Predictor	β (standardized)	SE	t	P-value	95% CI for β
(Intercept)	—	0.12	5.10	< 0.001	[0.98, 1.45]
Foundational Knowledge (F1)	0.08	0.05	1.60	0.11	[–0.01, 0.17]
Therapeutic Skills (F2)	0.35	0.04	8.75	< 0.001	[0.27, 0.43]
Problem Behavior Management (F3)	0.05	0.05	1.00	0.32	[–0.04, 0.14]
Multidisciplinary Collaboration (F4)	0.12	0.05	2.05	0.041	[0.01, 0.23]
Years of Clinical Experience	0.22	0.09	2.50	0.013	[0.05, 0.39]

Dependent variable: Overall satisfaction (0–10), N = 420; R² = 0.300; F(5, 414) = 35.49, p < 0.001. SE, standard error; CI, confidence interval; OLS, ordinary least squares.

The final model was statistically significant (F (5, 414) = 35.49, p < 0.001), accounting for 30.0% of the variance in satisfaction scores (R² = 0.30). We next examined between-profession differences using one-way ANOVA. ANOVA results by professional domain are summarized in **Table 5**.

**Table 5:** One-way analysis of variance of factor scores by professional domain.

Factor	F (2, 420)	p-value	*Post-hoc* (Bonferroni)
Foundational Knowledge (F1)	1.12	0.33	Ns
Therapeutic Skills (F2)	6.78	< 0.01	Clinical Care > Psychosocial Support (p < 0.05)
Problem Behavior Management (F3)	4.05	0.02	Education > Psychosocial Support (p < 0.05)
Multidisciplinary Collaboration (F4)	5.12	0.006	Education > Clinical Care (p < 0.05)

Dependent variables: Factor scores F1–F4 Professional domains: 1 = Clinical Care, 2 = education, 3 = Psychosocial Support; N = 423.

### 3.8 Coefficient estimates

- Therapeutic skills (F2) was the strongest predictor of satisfaction (β = 0.35, SE = 0.04, t = 8.75, p < 0.001), indicating that a +1 SD increase in F2 corresponded to a +0.35 SD increase in satisfaction.
- Years of clinical experience also showed a significant positive association (β = 0.22, SE = 0.09, t = 2.50, p = 0.013), suggesting that experienced professionals tend to report higher satisfaction.
- Multidisciplinary collaboration (F4) had a modest but significant effect (β = 0.12, SE = 0.05, t = 2.05, p = 0.041).
- Foundational knowledge (F1) and problem behavior management (F3) were not statistically significant predictors in this model.

Standardized regression coefficients are presented in **Table 6**.

**Table 6:** Multidisciplinary collaboration (F4).

Professional subgroup	N	Team-based care in child psychiatry	Inpatient treatment	Inpatient nursing	Dedicated psychology/MHSW roles
Clinical care	346	8.35 ± 2.01	8.12 ± 2.15	7.43 ± 2.56	7.81 ± 2.30
Education	18	6.78 ± 2.59	7.44 ± 2.48	5.22 ± 3.05	7.33 ± 2.31
Psychosocial Support	62	8.97 ± 1.52	8.53 ± 1.80	7.52 ± 2.05	9.02 ± 1.69
Total	426	8.37 ± 2.02	8.15 ± 2.13	7.35 ± 2.56	7.96 ± 2.27

Mean ratings (M ± SD) by professional subgroup.

MHSW:Mental Health Social Worker.

The model diagnostics confirmed that the key assumptions of the OLS regression were met. Residuals were approximately normally distributed (Shapiro–Wilk W = 0.99, p = 0.12), homoscedasticity was confirmed (Breusch–Pagan test χ² = 2.10, p = 0.35), and all VIFs were below 2.0, indicating no multicollinearity concerns.

## 4 Discussion

Building on our comprehensive results across the six domains, we can articulate both the need for enhanced multidisciplinary training and the specific curricular elements required.

### 4.1 Demographics and professional background

A wide range of professionals, psychiatrists and clinical psychologists, nurses, social workers, and educators, underscores the necessity of a domain-tailored curriculum. Clinical care professionals (psychiatrists and psychologists) and psychosocial support staff (social workers and occupational therapists) have distinct expertise and learning needs, while educational professionals require school-focused strategies. Therefore, future programs must include modular tracks that address each domain’s core competencies to ensure relevance and engagement by all participants.

### 4.2 Lecture-module ratings

**Figure 1:**
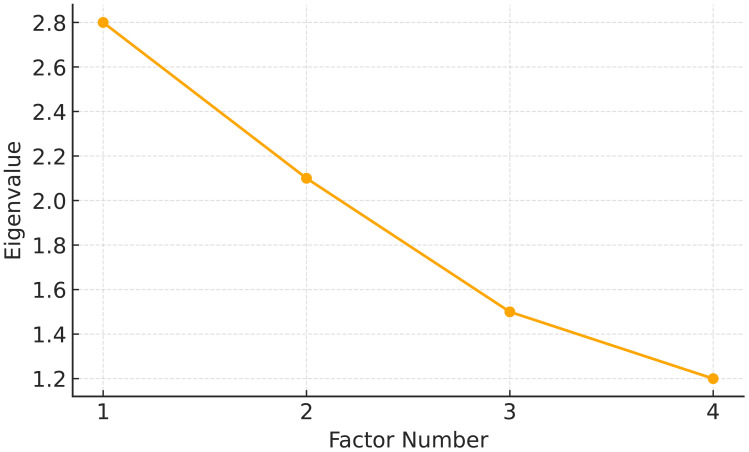
Scree plot from exploratory factor analysis. The scree plot shows the eigenvalues associated with each factor extracted from the 22 training modules. The sharp decline after the fourth component supported the selection of a four-factor solution.

EFA using maximum likelihood estimation was performed on the 22-lecture module ratings (N = 426) to identify the latent content domains. Four factors were extracted, as illustrated in **Figure 1**, which presents a scree plot supporting the four-factor solution.

Foundational modules (including disorder-specific lectures and welfare law) receive a consistently high NPS and should remain central. In contrast, low scores for sleep hygiene, play therapy, and electroconvulsive therapy highlighted areas for pedagogical redesign, shifting from didactic lectures to interactive workshops and skill labs. Therapeutic skills modules with moderate NPSs (including CBT and group psychotherapy) would benefit from case demonstrations and supervised practices.

**Figure 2:**
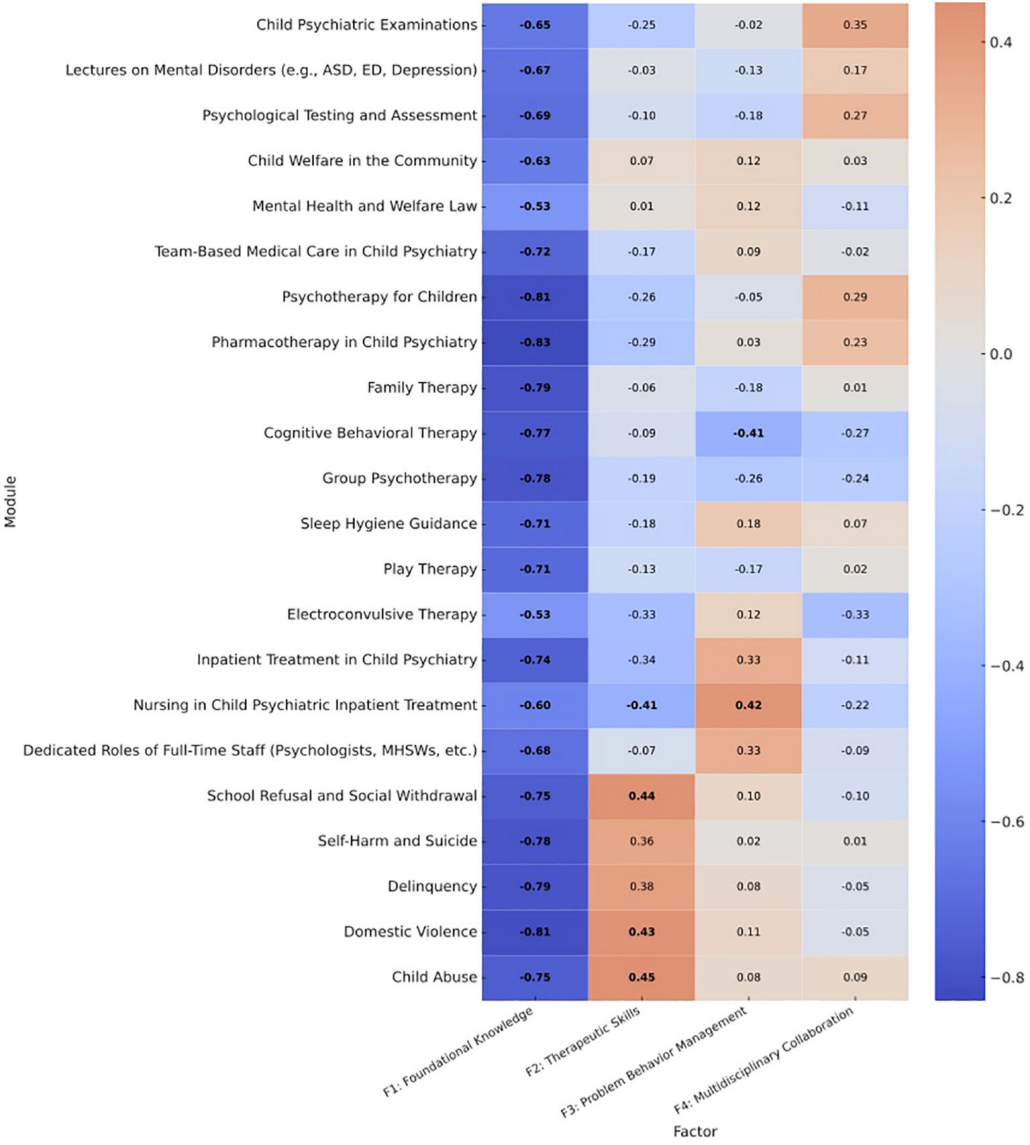
Heatmap of factor loadings for the 22 training modules. Factor loadings were derived from an exploratory factor analysis using principal axis factoring with varimax rotation. Cell values represent standardized loadings; boldface indicates primary loadings used for interpretation (|loading| ≥ 0.40). Note: The diverging color scale reflects both positive (red) and negative (blue) associations; lighter shades indicate stronger absolute loadings, whether positive or negative. Factor labels are:F1 = Foundational Knowledge, F2 = Therapeutic Skills, F3 = Problem Behavior Management, F4 = Multidisciplinary Collaboration. ASD, autism spectrum disorder; ED, Eating Disorder; MHSWs, Mental Health Social Workers.

Four factors were extracted: foundational knowledge (F1), therapeutic skills (F2), problem behavior management (F3), and multidisciplinary collaboration (F4), based on eigenvalues > 1.0 and scree plot inspection. The module loadings for each factor are shown in **Figure 2**, which is a heatmap of the factor loadings across the 22 modules.

### 4.3 Overall satisfaction

The strong average satisfaction (M = 7.4/10) and its drivers (therapeutic skills, clinical experience, and collaboration) indicate that programs emphasizing practical applicability and team-based exercises have maximum participants’ buy-in. Enhancing hands-on components and grouping participants by experience level (including novice vs. veteran clinicians) can further boost satisfaction.

### 4.4 Collaboration practices

With nearly one-third of facilities holding no regular multidisciplinary meetings and significant proportions of physicians and psychiatrists “never” attending, there is a clear gap in institutional collaboration. Training must incorporate structured meeting formats such as simulated ward case conferences and interagency panels, and teach information-sharing protocols to break down existing silos.

### 4.5 Follow-up programs

The majority (57%) favored annual refresher courses, suggesting that a continuous, rather than one-off, training model will sustain learning gains. Embedding short, recurring micro-learning sessions or digital “booster” modules between annual in-person events can maintain momentum and reinforce skills.

### 4.6 Open-ended feedback

As reflected in the open-ended responses, the participants expressed strong preferences for case-based learning, practical workshops (such as simulation and role-play), hybrid delivery formats, and iterative feedback systems. They also requested broader coverage of topics, such as trauma-informed care and school-based interventions.

Taken together, these insights mandate a competency-based blended learning curriculum that retains high-value foundational lectures in e-learning formats; transforms low-NPS modules into interactive workshops, clinical rotations, and simulation laboratories; embeds multidisciplinary case conferences as a core training modality; delivers annual refresher programs augmented by digital learning; and iterative evaluation loops to adapt content dynamically.

By aligning the training structure and content with the six domains of our survey, the revised syllabus will more effectively equip Japan’s diverse child and adolescent mental health workforce with integrated, high-quality care.

Notably, the revised training curriculum coincided with a major policy milestone: the inclusion of multidisciplinary training for non-physician staff in the FY2024 national insurance revision. This change formally recognized the role of allied health professionals in team-based psychiatric care as reimbursable under Japan’s healthcare system. The incorporation of inpatient care modules tailored to psychiatric nurses, clinical psychologists, and social workers reflects not only clinical needs but also direct responses to policy incentives. These findings suggest that structured multidisciplinary training can serve as a clinical and policy lever to improve care delivery and resource allocation in child and adolescent psychiatry.

The findings should also be interpreted within Japan’s cultural context, where professional hierarchies and consensus-oriented communication may shape interprofessional collaboration. Nevertheless, the observed emphasis on teamwork and role-based learning appears broadly applicable across other healthcare systems. The title phrase “From Silos to Synergy” reflects the observed transition from profession-specific, isolated learning structures to integrated, team-based approaches. This shift underscores the growing recognition that collaborative competencies are central to effective child psychiatric care in Japan.

These findings suggest that multidisciplinary training not only improves participant satisfaction and collaboration readiness but may also align with broader health policy trends. The recent inclusion of allied health training in the national insurance system underscores the strategic importance of equipping non-physician staff with specialized skills in child psychiatry.

### 4.7 Limitations

This study relied exclusively on post-training evaluation, which limited the ability to assess actual skill acquisition and behavioral change.

Future research should adopt a pre–post–follow-up design, integrating both quantitative assessments and qualitative feedback to evaluate learning retention and real-world impact.The response rate (34%) may have introduced sampling bias, as those more engaged or satisfied with the training could have been more likely to respond. Future studies should compare respondent and non-respondent profiles to enhance representativeness. The limitations of this study include its cross-sectional design, self-report measures, and aggregated domain classification, which may obscure the nuances among specific professions. Future longitudinal research with objective assessments and broader sampling is warranted.

Originally developed for business use, the NPS has also been used in health profession education as a practical indicator of trainee satisfaction (16–18).

This study did not conduct formal pilot testing or calculate internal consistency metrics (such as Cronbach’s α) for the 22 lecture‐module ratings or the derived NPSs. Consequently, the psychometric properties of our measurement instruments remain unverified, which potentially affects the precision of the domain constructs.

We did not specify procedures for managing incomplete or missing responses (such as listwise deletion or imputation). The absence of a documented missing‐data strategy may introduce bias into factor analysis, ANOVA, and regression results, and limit reproducibility.

No comparison was made between survey respondents and non-respondents regarding key demographics or professional characteristics. Consequently, the sample may overrepresent individuals with greater engagement or satisfaction, thereby reducing the generalizability of the findings to a broader trainee population.

The advanced program’s small-group case supervision component lacks clearly defined quantitative evaluation criteria in its source report. However, its impact was assessed only through qualitative feedback, thereby preventing an objective analysis of its effectiveness.

Future research should incorporate rigorous instrument validation, transparent handling of missing data, assessment of non-response bias, and quantitative metrics for in-person training components to strengthen the evidence base for multidisciplinary training interventions.

## 5 Conclusion

This study offers a data-driven foundation for designing a competency- and evidence-based training curriculum for child and adolescent psychiatry. By prioritizing practical therapeutic training, structured interprofessional collaborative exercises, and domain-tailored content, the proposed curriculum can better equip Japan’s multidisciplinary workforce to address the escalating needs of youth mental health care.

## Data Availability

The datasets used in this study are not publicly available. Because the data originates from a government survey, only materials publicly released by the government can be accessed. Requests for access to the datasets may be directed to usami.m@jihs.go.jp.
